# Effects of Ar Ion Irradiation on Mechanical Properties and Microstructure of SA508 Grade 3 Class 1 and Class 2 Reactor Pressure Vessel Steels

**DOI:** 10.3390/ma18194601

**Published:** 2025-10-03

**Authors:** Ho-A Kim, Mincheol Kim, Sungjun Choi, Sangtae Kim

**Affiliations:** 1 Department of Nuclear Engineering, Hanyang University, 222 Wangsimni-ro, Seongdong-gu, Seoul 04763, Republic of Korea; pissan@hanyang.ac.kr (H.-A.K.); sjchoi98@hanyang.ac.kr (S.C.); 2 Department of Materials Science and Engineering, Hanyang University, 222 Wangsimni-ro, Seongdong-gu, Seoul 04763, Republic of Korea

**Keywords:** reactor pressure vessel steels, ion irradiation, mechanical tests, nanoindentation, transmission electron microscopy

## Abstract

This study investigates the effects of Ar ion irradiation on the mechanical properties and microstructure of SA508 Grade 3 Class 1 and Class 2 reactor pressure vessel steels. Three different fluence levels of Ar ion irradiation were applied to simulate accelerated irradiation damage conditions. Charpy impact and tensile tests conducted before and after irradiation showed no significant changes in bulk mechanical properties. Stopping and Range of Ions in Matter (SRIM) and Transport of Ions in Matter (TRIM) simulations revealed that Ar ion irradiation produces a shallow penetration depth of approximately 2.5 µm, highlighting the limitations of conventional macro-mechanical testing for evaluating irradiation effects in such a thin surface layer. To overcome this limitation, nano-indentation tests were performed, revealing a clear increase in indentation hardness after irradiation. Transmission electron microscopy (TEM) analysis using STEM–BF imaging confirmed a higher density of irradiation-induced defects in the irradiated specimens. The findings demonstrate that while macro-mechanical properties remain largely unaffected, micro-scale testing methods such as nano-indentation are essential for assessing irradiation-induced hardening in shallowly damaged layers, providing insight into the behavior of SA508 reactor pressure vessel steels under accelerated irradiation conditions.

## 1. Introduction

Reactor pressure vessel (RPV) steels are subjected to severe neutron irradiation during reactor operation, resulting in notable material degradation phenomena such as irradiation embrittlement [1,2]. This embrittlement is primarily associated with the formation of nanometer-scale defects—vacancy clusters, dislocation loops, and copper-rich precipitates—which impede dislocation motion and lead to increased hardness and a higher ductile-to-brittle transition temperature (DBTT) [3,4]. In the case of SA508 Grade 3 steel, neutron collisions generate displacement cascades, producing primary knock-on atoms that create a complex population of irradiation-induced defects [3]. The extent of displacement damage is commonly quantified by the displacement per atom (dpa) metric, providing a standardized basis for evaluating the intensity of irradiation exposure across different experimental platforms [5]. This mechanistic understanding forms the foundation of efforts to evaluate and improve RPV material longevity in advanced reactors, particularly for Korea’s integrated small modular reactor (i-SMR) program which targets an extended design lifetime of 80 years and involves transition from SA508 Grade 3 Class 1 to Class 2 steel for RPV construction [6,7]. Because Class 1 and Class 2 variants differ in nickel and manganese contents—which govern hardenability, carbide precipitation behavior, and ultimately irradiation sensitivity—their direct comparison under controlled Ar ion irradiation provides critical insight into how alloy chemistry influences defect evolution, hardening kinetics, and embrittlement propensity in reactor pressure vessel steels.

Conventional experimental approaches to studying neutron irradiation effects have relied on test reactor campaigns, which enable in situ exposure of structural materials to reactor-relevant neutron fluxes [2]. However, these experiments are constrained by lengthy irradiation durations—often spanning years to decades—to achieve damage accumulations equivalent to extended reactor service lifetimes, owing to relatively low damage rates on the order of 10^−7^ to 10^−6^ dpa/s [4]. The resultant radioactivity of neutron-irradiated specimens introduces significant logistical and financial burdens on post-irradiation examination, further hindering the systematic study of advanced RPV materials for next-generation reactors. These limitations necessitate alternative approaches for efficiently predicting material degradation in the face of increasingly challenging design lifetimes and safety requirements [4].

Heavy ion irradiation has thus gained wide adoption as a surrogate method for replicating neutron irradiation damage within laboratory settings [2,8]. Ion beams can achieve damage rates orders of magnitude higher than neutron sources, enabling rapid attainment of high dpa levels analogous to end-of-life reactor conditions within days to weeks [2,4]. Ion irradiation also eliminates the problem of sample activation and allows for precise control over experimental variables such as temperature, irradiation dose, and dose rate [2]. Numerous comparative studies have demonstrated that heavy ion irradiation, when calibrated correctly for damage equivalency and microstructural features, can reliably emulate many aspects of neutron-induced damage such as defect clustering and irradiation hardening [9,10]. The use of the dpa metric, computed via simulation tools such as SRIM/TRIM, is well-established for cross-referencing damage levels between ion and neutron experiments [11,12]. However, a critical limitation of heavy ion irradiation is the extremely shallow penetration of incident ions; for example, Ar ions at commonly used energies produce a damage depth of only 2–3 μm, as predicted by SRIM/TRIM simulation [12,13,14]. As a result, mechanical property modifications are generally confined to this near-surface region, making conventional macro-mechanical tests like Charpy impact and tensile testing insensitive to ion-induced property changes [15].

To overcome this intrinsic depth limitation, micro- and nano-scale mechanical testing methods have been integrated into irradiation research protocols, with nanoindentation emerging as a particularly powerful technique for characterizing irradiated layers with micro-scale depth resolution [14,16,17]. Nanoindentation allows direct, quantitative measurement of hardness and modulus within the irradiated zone, revealing hardening trends that are spatially coincident with the damage profile yielded by ion irradiation [18,19]. By combining nanoindentation with transmission electron microscopy (TEM) studies, we can correlate observed mechanical hardening with specific microstructural features such as defect density, size, and distribution within the irradiated layer [9]. These methods collectively enable effective assessment of irradiation-induced degradation in RPV steels, offering indispensable insights for materials selection and life prediction in nuclear applications where macro-scale test methods fail to capture localized irradiation effects [9,16].

## 2. Experimental Methods

### 2.1. Materials

SA508 Grade 3 Class 1 and Class 2 low alloy steels, widely used for nuclear reactor pressure vessel applications, were provided by Doosan Enerbility, Changwon, South Korea for this study. According to ASME specifications [20] and prior characterization reports [21], SA508 Grade 3 Class 1 steel typically contains approximately 0.21 wt.% C, 1.36 wt.% Mn, 0.92 wt.% Ni, 0.21 wt.% Cr, and 0.49 wt.% Mo. In contrast, SA508 Grade 3 Class 2 steel exhibits a slightly higher carbon content at 0.24 wt.%, with 1.39 wt.% Mn, 0.86 wt.% Ni, 0.22 wt.% Cr, and 0.53 wt.% Mo. For specimen preparation, large blocks of material were sectioned and precisely machined into Charpy impact and tensile specimens using electrical discharge machining (EDM). This manufacturing approach was chosen to minimize thermal and mechanical alteration, such as work hardening, near the cut surfaces. After EDM processing, all specimens were thoroughly cleaned in an ultrasonic bath, using acetone followed by ethanol, to ensure the removal of fine particulates and any residual dielectric fluid from machining.

### 2.2. Ar Ion Irradiation

**Figure 1:**
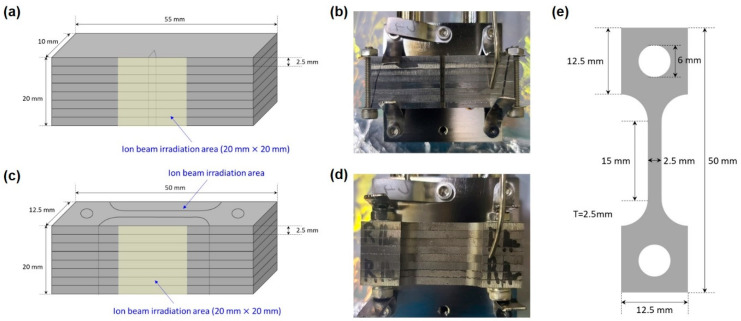
(**a**) Stacking arrangement and ion beam irradiation area (20 mm × 20 mm) for Charpy impact specimens, (**b**) photograph of the actual Charpy specimen irradiation setup, (**c**) stacking arrangement and ion beam irradiation area (20 mm × 20 mm) for tensile specimens, (**d**) photograph of the actual tensile specimen irradiation setup, and (**e**) schematic of the tensile specimen geometry.

Ar ion irradiation was conducted at the Korea Atomic Energy Research Institute’s Heavy Ion Beam Facility (KAHIF), a facility developed in-house at KAERI, located in Daejeon, South Korea. A 6.871 MeV Ar^2+^ beam was raster-scanned over a 20 × 20 mm^2^ area on each specimen surface at room temperature under high vacuum (<1 × 10^−5^ Pa). Average ion flux was 2.8 × 10^12^ ions/cm^2^/s. Three fluence levels—1.684 × 10^15^, 1.010 × 10^16^, and 2.021 × 10^16^ ions/cm^2^—were selected to cover low-to-high damage regimes, delivered over irradiation durations of approximately 10 min, 1 h, and 2 h, respectively. Dose rate, total fluence, and temperature were continuously monitored and recorded to ensure reproducibility and to support correlation with observed damage evolution. Ar ion irradiation was performed on both Charpy impact and tensile specimens. To match the ion beam spot size of 20 × 20 mm^2^, four SA508 Grade 3 Class 1 specimens and four SA508 Grade 3 Class 2 specimens were stacked and irradiated together for each test type. The schematic setup of ion irradiation for the Charpy specimens is shown in Figure 1a, with an actual photo of the irradiation experiment presented in Figure 1b. Similarly, the tensile specimen irradiation setup is illustrated in Figure 1c, and its corresponding photo is provided in Figure 1d. To estimate the ion penetration depth and damage profile, Monte Carlo simulations were performed using the SRIM software (version 2013), developed by J.F. Ziegler at the IBM Research Division, Yorktown Heights, New York, USA. The TRIM module within SRIM was specifically used. The simulation was setup with the experimentally determined chemical composition of SA508 Grade 3 Class 2 steel as the target. The incident ion type (Ar), energy (6.871 MeV), and fluence were matched to the experimental conditions. The quick Kinchin–Pease model was used to estimate atomic displacements (dpa) as a function of depth. For each simulation, 10,000 ions were tracked to ensure statistical reliability. The outputs included the depth distribution of implanted ions, the spatial profile of displacement damage, and the lateral and longitudinal straggling of the ion trajectories. All simulations assumed an amorphous target and did not account for dynamic composition changes or crystallographic effects, consistent with the standard SRIM/TRIM approach.

### 2.3. Mechanical Testing

Charpy impact tests were carried out on subsize specimens measuring 10 × 2.5 × 55 mm according to ISO 148-1 [22], using an instrumented pendulum impact tester (Model KDPI-180, KD Precision Co., Ltd., Seoul, Republic of Korea). Due to time and cost constraints of ion irradiation, each condition (irradiation fluence and steel variant) was tested with two replicate specimens. The tensile specimen geometry is shown in Figure 1e. Tensile tests were carried out on specimens with a gauge length of 15 mm in an Instron 5582 testing frame equipped with a high-temperature furnace. Specimens were heated at 5 °C min^−1^ to 300 °C, held for 30 min to achieve thermal equilibrium, and then strained at a constant strain rate of 1 × 10^−3^ s^−1^ until fracture, with two replicates per condition. Nanoindentation tests were performed directly on the irradiated specimen surface using a Berkovich diamond tip. For each condition, twenty indents per specimen were performed with at least 10 µm spacing to avoid interaction of plastic zones. Indentation depth was limited to a maximum of 200 nm, which is less than 10% of the irradiated damage layer thickness, to ensure accurate measurement within the damaged zone [23,24,25]. Hardness and reduced modulus were derived from load–displacement curves using the Oliver–Pharr method [26]. All mechanical data (impact energy, yield strength, ultimate tensile strength, total elongation, hardness, and reduced modulus) are presented as mean ± standard deviation and plotted in the corresponding figures.

### 2.4. Microstructural Analysis

Cross-sectional TEM specimens were prepared from the irradiation-affected regions using focused ion beam (FIB) milling on a Helios G5 UC instrument (Thermo Fisher Scientific, Waltham, MA, USA). The FIB technique allowed precise extraction of electron-transparent lamellae, typically thinned to around 100 nm thickness for optimal imaging conditions. TEM observations were carried out at 200 kV accelerating voltage using a JEOL JEM 2100F microscope (JEOL Ltd., Tokyo, Japan). Bright-field scanning transmission electron microscopy (STEM-BF) imaging was employed to visualize irradiation-induced defects such as dislocation loops and vacancy clusters [27,28,29,30,31]. Selected area electron diffraction (SAED) patterns were also acquired to assess crystallographic changes and to confirm irradiation-induced lattice distortions [32,33,34,35]. This microstructural information was correlated with mechanical testing results to understand damage mechanisms in SA508 Grade 3 Class 1 and Class 2 steels.

## 3. Results

### 3.1. SRIM/TRIM Simulation Results

Prior to the ion irradiation experiments, SRIM/TRIM simulations were carried out to predict the damage profile under the specific test conditions. The simulations used the chemical composition of SA508 Grade 3 Class 2 steel and the 6.871 MeV Ar ion beam available at the KAHIF. Only the Class 2 composition was used as a reference for both simulation and experimental planning, as the minor compositional differences between Class 1 and Class 2 steels were not expected to significantly affect the results. These results were used to select appropriate indentation depths for nanoindentation testing and to interpret the relationship between mechanical property changes and the simulated damage distribution.

**Figure 2:**
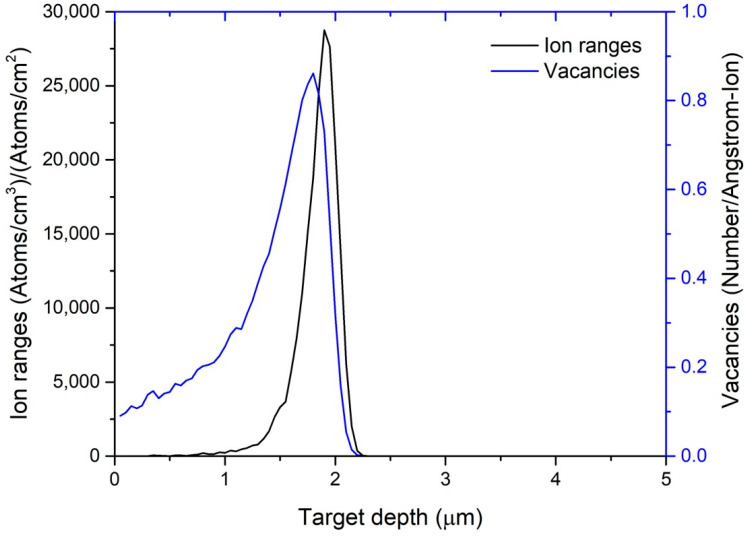
SRIM/TRIM simulation results for 6.871 MeV Ar^2+^ irradiation in SA508 Grade 3 Class 2: the black curve shows the depth profile of implanted ion range (atoms·cm^−2^ per µm) and the blue curve shows the depth-dependent vacancy production rate (vacancies per ion per Å).

The results, summarized in Figure 2, show that more than 90% of displacement damage is confined to a narrow surface layer between 1.8 µm and 2.2 µm depth. The projected ion range of 1.81 µm and a lateral straggle of 1970 Å indicate that while ions remain highly localized in depth, there is modest lateral spread within the plane. Vacancy concentration peaks at approximately 1.7 µm depth, reaching over 1 × 10^22^ atoms·cm^−3^ per ion, whereas the collision event rate (vacancies per ion per Å) maximizes at the same depth, reflecting dominant nuclear stopping in this terminal region. Beyond ~2.2 µm, both vacancy and collision profiles drop to near zero, confirming negligible damage deeper in the matrix.

These localized damage profiles directly informed the selection of nanoindentation parameters: indentation depths were restricted to less than 200 nm (≈10% of the damage layer thickness) to ensure sampling solely the irradiated zone, and multiple indents were performed across the irradiated area to account for lateral straggle-induced variability. Furthermore, the simulated vacancy densities were converted to displacement per atom (dpa) values, enabling precise calculation of the ion fluences required to achieve target dpa levels in the experimental specimens and thereby correlating mechanical property changes with known damage doses.

### 3.2. Macro-Scale Mechanical Test Results

Macro-scale mechanical testing was conducted to assess the effect of Ar ion irradiation on the bulk properties of SA508 Grade 3 Class 1 and Class 2 steels. Charpy impact tests and uniaxial tensile tests were performed on specimens subjected to progressively higher ion fluence (1.684 × 10^15^, 1.010 × 10^16^, and 2.021 × 10^16^ ions/cm^2^), enabling the evaluation of irradiation-induced changes in toughness, yield strength, and ultimate tensile strength at the structural scale.

**Figure 3:**
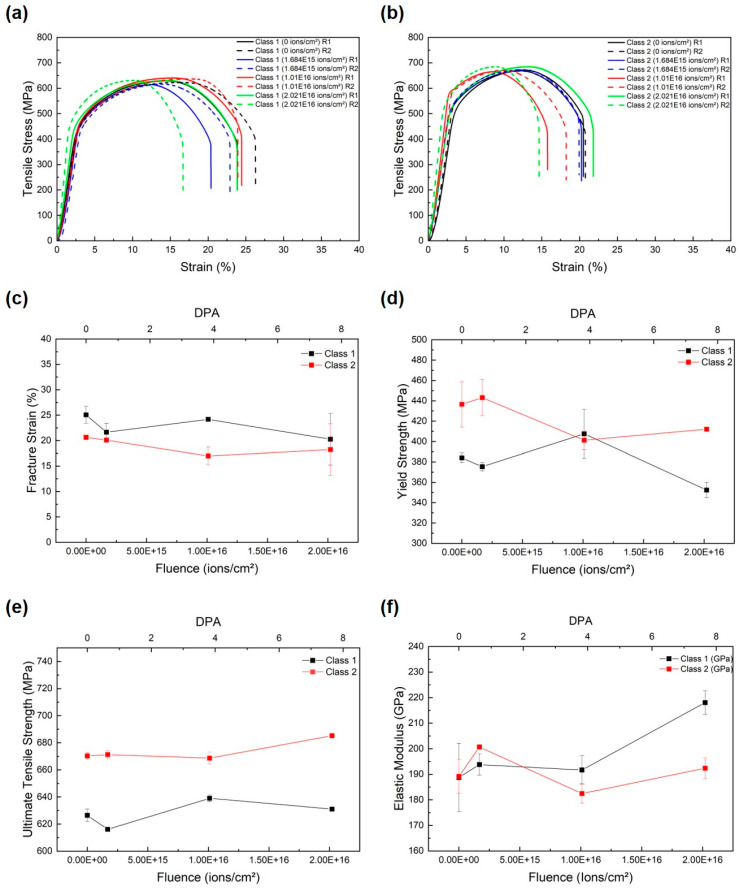
Mechanical properties of SA508 Grade 3 Class 1 and Class 2 steels as a function of Ar ion fluence (dpa): (**a**) tensile stress–strain curves for Class 1, (**b**) tensile stress–strain curves for Class 2, (**c**) fracture strain versus fluence, (**d**) yield strength versus fluence, (**e**) ultimate tensile strength versus fluence, and (**f**) elastic modulus versus fluence.

Figure 3a,b present the true tensile stress–strain curves for Class 1 and Class 2, respectively. Both grades exhibit typical ferritic–bainitic behavior with an initial linear elastic region followed by yield and strain hardening. Upon irradiation, the overall shape of the stress–strain curves remains consistent, with shifts in the yield point and minor changes in the post-yield slope. Notably, the strain at which necking initiates shows slight variation among fluence levels, particularly in Class 1 where the curves for the two highest fluences (1.010 × 10^16^ and 2.021 × 10^16^ ions/cm^2^) converge toward reduced elongation. Figure 3c plots fracture strain versus ion fluence. Class 1 displays fracture strains of 25.1% at 0 ions/cm^2^ (unirradiated), 21.6% at 1.684 × 10^15^ ions/cm^2^, 24.2% at 1.010 × 10^16^ ions/cm^2^, and 20.3% at 2.021 × 10^16^ ions/cm^2^. Class 2 shows a more uniform decline: 20.6%, 20.1%, 17.0%, and 18.2% at the respective fluences. Fluctuations in fracture strain are apparent in both grades, but the overall trend indicates a modest reduction in ductility with increasing fluence.

Figure 3d shows yield strength as a function of fluence. For Class 1, yield strength starts at 383 MPa (unirradiated), decreases to 374 MPa at 1.684 × 10^15^ ions/cm^2^, peaks at 408 MPa at 1.010 × 10^16^ ions/cm^2^, and then declines to 354 MPa at 2.021 × 10^16^ ions/cm^2^. Class 2 begins at 438 MPa, increases slightly to 442 MPa at 1.684 × 10^15^ ions/cm^2^, drops to 401 MPa at 1.010 × 10^16^ ions/cm^2^, and recovers to 413 MPa at 2.021 × 10^16^ ions/cm^2^. These non-monotonic variations remain within the typical ± 20 MPa scatter observed for RPV steel tensile tests. Figure 3e depicts ultimate tensile strength (UTS) versus fluence. Class 1 UTS varies from 627 MPa (unirradiated) to 618 MPa at 1.684 × 10^15^ ions/cm^2^, rising to 639 MPa at 1.010 × 10^16^ ions/cm^2^ before settling at 633 MPa at 2.021 × 10^16^ ions/cm^2^. Class 2 maintains higher UTS values overall, ranging from 670 MPa to 684 MPa, with a slight dip at 1.010 × 10^16^ ions/cm^2^ and subsequent increase at the highest fluence.

Figure 3f presents elastic modulus derived from the initial linear portions of the stress–strain curves. Class 1 exhibits modulus values of 189 GPa (unirradiated), 195 GPa (1.684 × 10^15^ ions/cm^2^), 192 GPa (1.010 × 10^16^ ions/cm^2^), and 218 GPa (2.021 × 10^16^ ions/cm^2^). Class 2 shows 189 GPa, 200 GPa, 182 GPa, and 193 GPa at the corresponding fluences. While Class 2’s modulus fluctuates without a clear trend, Class 1 demonstrates a pronounced increase at the highest fluence.

**Figure 4:**
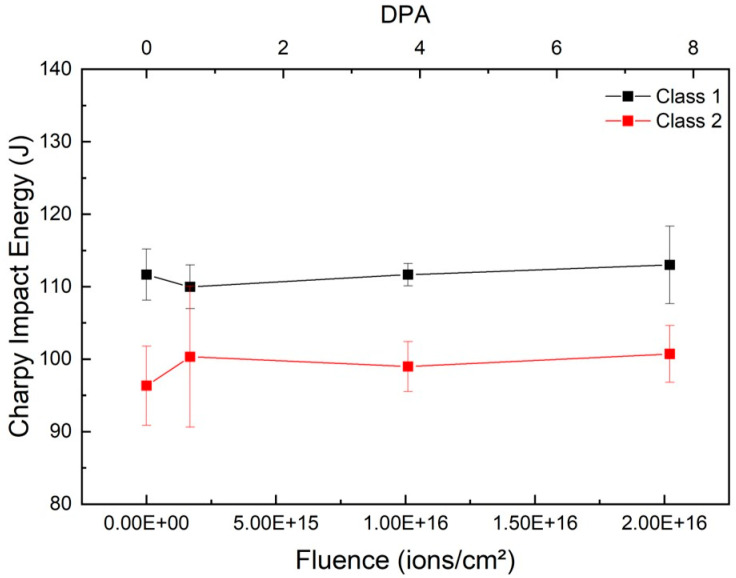
Charpy impact energy of SA508 Grade 3 Class 1 and Class 2 steels as a function of Ar ion fluence.

Figure 4 shows the Charpy V-notch impact energy of SA508 Grade 3 Class 1 and Class 2 steels as a function of Ar ion fluence. Class 1 exhibits an average impact energy of 112 J in the unirradiated condition, which decreases slightly to 110 J at 1.684 × 10^15^ ions/cm^2^ returns to 112 J at 1.010 × 10^16^ ions/cm^2^, and increases to 113 J at 2.021 × 10^16^ ions/cm^2^. Class 2 starts at 96 J, rises to 100 J at 1.684 × 10^15^ ions/cm^2^, dips to 99 J at 1.010 × 10^16^ ions/cm^2^, and recovers to 101 J at the highest fluence. The variations in both grades remain within ±3 J (≈3%) of their unirradiated baselines, indicating that bulk fracture toughness is essentially unchanged by the shallow (~2.2 µm) irradiation damage layer.

Overall, macro-scale tests reveal only modest, non-systematic changes in strength and toughness with increasing ion fluence, alongside a slight downward trend in ductility. Both Class 1 and Class 2 retain similar tensile stress–strain behavior, with yield strength and UTS varying within ±20 MPa, elastic modulus showing only a pronounced increase in Class 1 at the highest fluence, and Charpy impact energy changing by less than 3%. Fracture strain decreases from 25.1% to 20.3% for Class 1 and from 20.6% to 18.2% for Class 2 across the fluence range, indicating a modest loss of ductility. These findings confirm that conventional bulk mechanical measurements exhibit limited sensitivity to the shallow (~2.2 µm) irradiation damage layer predicted by SRIM/TRIM simulations.

### 3.3. Nanoindentation Test Results

**Figure 5:**
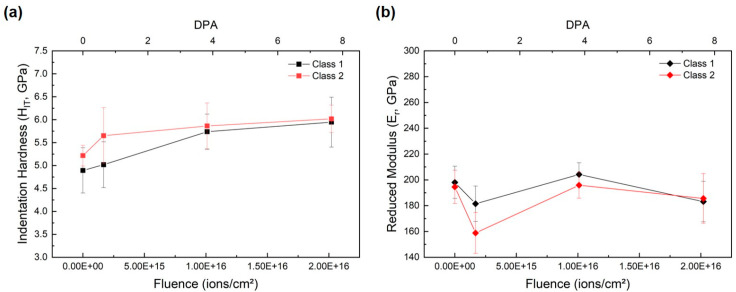
(**a**) Indentation hardness and (**b**) indentation modulus of SA508 Grade 3 Class 1 and Class 2 steels measured by nanoindentation as a function of Ar ion fluence.

To quantify irradiation-induced hardening confined to the near-surface region, nanoindentation tests were conducted on SA508 Grade 3 Class 1 and Class 2 specimens with indentation depths limited to ≤200 nm (≈10% of the damage layer thickness) based on SRIM/TRIM predictions. Indentation hardness (H_IT_) and reduced modulus (E_r_) were extracted from load–displacement curves using the Oliver–Pharr method, and their variation with ion fluence is plotted in Figure 5.

Figure 5a shows that the unirradiated hardness of Class 1 and Class 2 steels is 4.9 GPa and 5.2 GPa, respectively. Upon irradiation to 1.684 × 10^15^ ions/cm^2^, hardness increases to 5.0 GPa for Class 1 and to 5.6 GPa for Class 2. Further irradiation to 1.010 × 10^16^ ions/cm^2^ drives hardness to 5.7 GPa in Class 1 and 5.8 GPa in Class 2. At the highest fluence of 2.021 × 10^16^ ions/cm^2^, hardness reaches 5.9 GPa for Class 1 and 6.0 GPa for Class 2. Thus, Class 1 exhibits a 20.4% increase in hardness from its baseline, while Class 2 shows a 15.4% increase, indicating slightly stronger hardening in Class 1 despite its initially lower hardness.

Figure 5b presents the reduced modulus results. Unirradiated E_r_ values are 198 GPa for Class 1 and 193 GPa for Class 2. At 1.684 × 10^15^ ions/cm^2^, Class 1 modulus decreases to 182 GPa, and Class 2 drops to 158 GPa. Upon irradiation to 1.010 × 10^16^ ions/cm^2^, both grades recover: E_r_ increases to 203 GPa for Class 1 and to 196 GPa for Class 2. At the highest fluence, modulus returns toward baseline values (182 GPa for Class 1 and 185 GPa for Class 2). The non-monotonic fluctuations in E_r_—initial decrease, mid-fluence recovery, and stabilization—contrast with the monotonic H_IT_ increase, suggesting that plastic resistance is more sensitive to irradiation-induced defects than elastic stiffness.

These nanoindentation results demonstrate clear hardening within the shallow damage zone, with Class 1 steel experiencing a larger relative increase in hardness despite its initially lower absolute value. The more subdued and non-systematic changes in reduced modulus highlight that elastic properties are less directly governed by defect accumulation under these conditions.

### 3.4. TEM Microstructural Analysis

**Figure 6:**
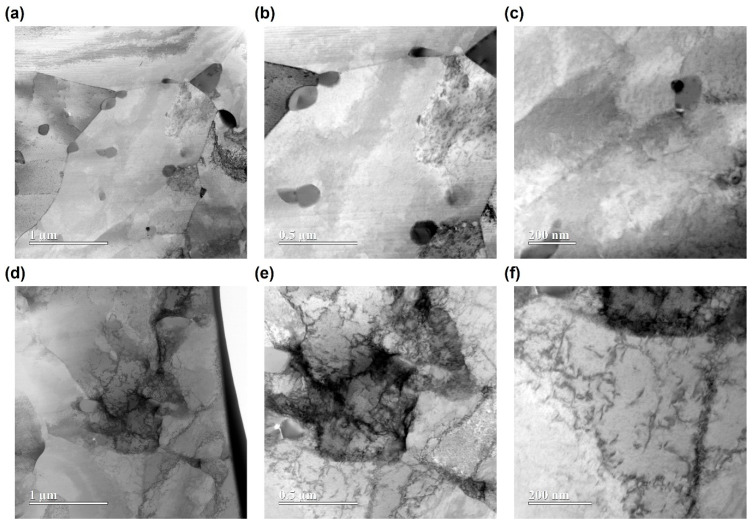
STEM-BF images of SA508 Grade 3 Class 1 before ((**a**–**c**), unirradiated) and after ((**d**–**f**), irradiated at 2.021 × 10^16^ ions/cm^2^) Ar ion irradiation at various magnifications.

**Figure 7:**
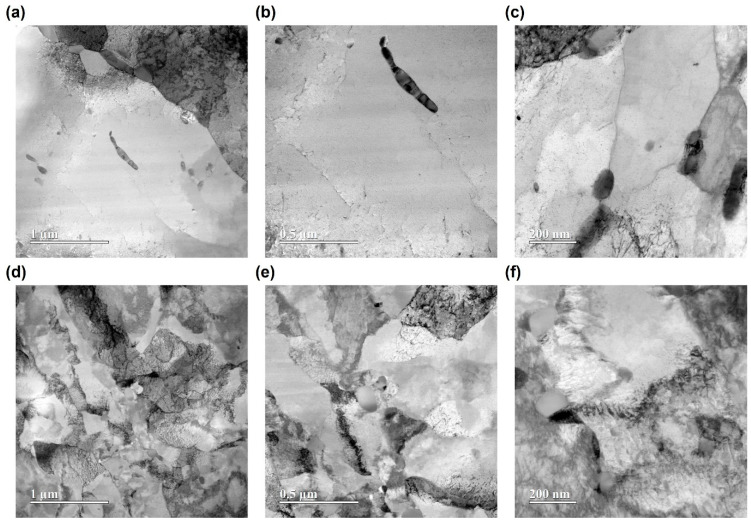
STEM-BF images of SA508 Grade 3 Class 2 before ((**a**–**c**), unirradiated) and after ((**d**–**f**), irradiated at 2.021 × 10^16^ ions/cm^2^) Ar ion irradiation at various magnifications.

The microstructural changes induced by Ar ion irradiation were systematically investigated using STEM-BF imaging and SAED pattern analysis. Figure 6 and Figure 7 provide a side-by-side comparison of the microstructures before and after irradiation for SA508 Grade 3 Class 1 and Class 2 steels, respectively, at various magnifications (from 1 μm to 200 nm).

For SA508 Grade 3 Class 1 steel, the unirradiated condition (Figure 6a–c) displays a heterogeneous microstructure with regions of varying contrast and several clearly visible, larger carbide precipitates. These features are well-distributed, and different regions can be distinguished by variations in image brightness and texture. After Ar ion irradiation to a fluence of 2.021 × 10^16^ ions/cm^2^ (Figure 6d–f), there is a pronounced increase in the density of small, dark contrast features throughout the matrix. These irradiation-induced defects, appearing as black dots, dislocation loops and clusters, are heterogeneously distributed; some regions show higher concentrations than others. At intermediate and higher magnifications (Figure 6e–f), individual defect clusters are resolved more clearly, and both isolated point-like defects and larger agglomerated features can be identified. Overall, the irradiated Class 1 steel exhibits a distinctly mottled appearance compared to its unirradiated state, while still retaining its core microstructural framework.

SA508 Grade 3 Class 2 steel shows comparable microstructural characteristics to Class 1 in the unirradiated condition (Figure 7a–c), with carbide precipitates and matrix regions displaying similar contrast. Subtle differences in carbide morphology and spatial distribution can be detected. However, after irradiation (Figure 7d–f), Class 2 steel displays an increase in dark contrast features, but the overall defect density and degree of clustering visible in the micrographs appear less pronounced than in Class 1. The defects are present and form clusters, but the background matrix remains comparatively less mottled, and the spatial distribution of defects tends to be more localized. At higher magnifications, clusters are visible, but they are generally less pervasive than those observed in Class 1 at the same irradiation conditions.

**Figure 8:**
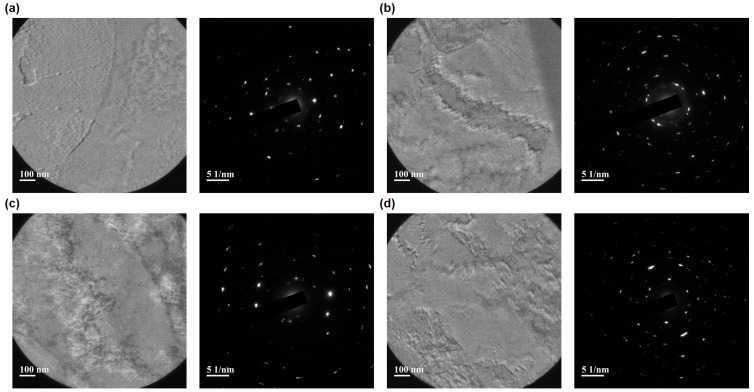
SAED patterns of SA508 Grade 3 Class 1 ((**a**), unirradiated; (**b**), irradiated) and Class 2 ((**c**), unirradiated; (**d**), irradiated) before and after Ar ion irradiation at 2.021 × 10^16^ ions/cm^2^.

SAED pattern analysis (Figure 8) further supports these observations. Both Class 1 (Figure 8a) and Class 2 (Figure 8c) unirradiated specimens show sharp, well-defined diffraction spots. The irradiation-induced changes are visible in both grades (Figure 8b,d); however, Class 1 shows a distinct increase in diffuse scattering and spot broadening compared to Class 2, corresponding to greater lattice distortion and defect accumulation.

The STEM-BF images and SAED patterns demonstrate that, under the given conditions, Class 1 develops a more abundant and widespread population of irradiation-induced defects compared to Class 2. This observation is consistent with mechanical results indicating greater irradiation hardening in Class 1. The more severe defect accumulation in Class 1 provides a microstructural explanation for its enhanced hardness increase upon irradiation, highlighting the direct correlation between defect formation and mechanical property evolution in the two grades.

## 4. Discussion

SRIM/TRIM simulations (Figure 2) demonstrate that 6.871 MeV Ar^2+^ ions produce a damage profile confined to the upper ~1.8–2.2 µm of the SA508 Grade 3 surface. Under these conditions, more than 90% of displacement damage is concentrated within this narrow region, with negligible damage beyond ~2.2 µm. Because Charpy and tensile specimens have millimeter-scale dimensions, the irradiated layer constitutes less than 0.2% of the total cross-sectional area. Consequently, macro-scale tests integrate responses from both the damaged surface and the pristine bulk, diluting any irradiation-induced modifications and rendering bulk tensile properties and impact energies insensitive to the localized damage [2,36].

Uniaxial tensile tests (Figure 3) show true stress–strain curves for both Class 1 and Class 2 steels that overlap closely across all fluences. Yield strength and ultimate tensile strength vary by no more than ± 20 MPa—within the expected scatter for RPV steels—and no systematic hardening or softening trend emerges. Elastic modulus remains near 190 GPa for most conditions, with only Class 1 exhibiting a pronounced increase to ~218 GPa at the highest fluence. Charpy impact energies (Figure 4) similarly fluctuate by less than 3% around their unirradiated baselines (112 J for Class 1; 96 J for Class 2). These macro-mechanical results confirm that the shallow irradiation damage layer exerts minimal influence on bulk material behavior. Despite the apparent insensitivity of bulk metrics, fracture strain exhibits a modest but consistent downward trend with fluence: Class 1 decreases from 25.1% to 20.3%, and Class 2 from 20.6% to 18.2%. This reduction in ductility reflects the early onset of irradiation embrittlement within the damaged surface [37]. Dislocation loops, vacancy clusters, and small defect agglomerates formed during ion irradiation act as obstacles to dislocation motion, reducing work-hardening capacity and strain to failure. However, because these features are confined to the near-surface region, their effect on macroscopic elongation is muted, appearing only as a slight decrease in overall fracture strain.

Nanoindentation (Figure 5) provides direct insight into mechanical changes within the irradiated zone. Indentation hardness increases monotonically with fluence, rising by 20.4% in Class 1 (4.9 → 5.9 GPa) and 15.4% in Class 2 (5.2 → 6.0 GPa). These hardening levels exceed typical experimental scatter and unequivocally demonstrate irradiation-induced strengthening [2,14]. The more pronounced hardening in Class 1, despite its lower initial hardness, suggests that Class 1 develops a higher density or larger volume fraction of irradiation defects that impede plastic flow. Reduced modulus exhibits non-monotonic variation—Class 1 drops from 198 GPa to 182 GPa at 1.684 × 10^15^ ions/cm^2^, recovers to 203 GPa at 1.010 × 10^16^ ions/cm^2^, and returns toward baseline (~182 GPa) at 2.021 × 10^16^ ions/cm^2^—indicating that elastic stiffness is less directly affected by defect accumulation than plastic hardness [23]. The findings presented in this study align with recent comprehensive reviews on nanoindentation testing of ion-irradiated materials, which emphasize the critical importance of addressing various technical challenges including surface effects, pile-up phenomena, and indentation size effects that can significantly impact measurement accuracy [38]. These methodological considerations are particularly relevant for shallow damage layers where precise control of indentation parameters becomes essential for obtaining reliable mechanical property data from the irradiated zone.

STEM–BF micrographs and SAED patterns (Figure 6, Figure 7 and Figure 8) reveal a denser population of nanometer-scale defect clusters, dislocation loops, and enhanced diffuse scattering in irradiated Class 1 compared to Class 2 [9]. These microstructural observations correlate with both the larger relative hardness increase and the elastic modulus rise in Class 1 under higher fluence. The localized hardening measured by nanoindentation and the subtle ductility loss in tensile tests can be directly attributed to the proliferation of irradiation-induced defects within the ~2 µm surface layer.

The increase in indentation hardness observed after irradiation can be interpreted using the Orowan mechanism [39], which describes how dispersed nanoscale obstacles—such as defect clusters and dislocation loops—impede dislocation motion. According to this model, the yield strength increment (∆σ) is inversely proportional to the average spacing between obstacles (λ):

(1) $$∆\sigma =M\alpha \frac{Gb}{\alpha }$$

where M is the Taylor factor, α the obstacle strength, G the shear modulus, and b the Burgers vector. In this study, TEM analysis revealed a higher density of defect clusters and loops in Class 1, resulting in a smaller *λ* and thus a larger increase in yield strength. Since indentation hardness is empirically proportional to yield strength ($H\approx C\cdot \sigma_{y}$), the greater hardness increase in Class 1 can be directly attributed to the more abundant irradiation-induced defects observed in TEM. This mechanistic link between microstructure and mechanical properties supports the conclusion that irradiation hardening in these steels is governed by the density and distribution of nanoscale defect obstacles.

Collectively, these multi-scale results underscore the limitations of conventional macro-mechanical testing for ion-irradiated specimens with shallow damage profiles. While bulk strength and toughness remain dominated by the pristine substrate, localized embrittlement and hardening are clearly manifested at the micro- and nano-scale. To fully characterize irradiation effects in RPV steels, future studies should integrate additional localized mechanical assays—such as micro-pillar compression, micro-cantilever bending, and in situ TEM deformation—alongside nanoindentation and high-resolution microscopy. This comprehensive, multi-length-scale approach will enable quantitative mapping of irradiation-induced changes in yield strength, fracture toughness, and strain localization within the damaged zone, thereby providing deeper mechanistic understanding of irradiation hardening and embrittlement processes in nuclear structural materials.

## 5. Conclusions

Ar ion irradiation at 6.871 MeV produced a highly localized damage profile in SA508 Grade 3 steels, with SRIM/TRIM simulations indicating a projected ion range of ~1.8 µm and a damage depth of ~2.2 µm. Because this damaged layer constitutes only a minute fraction of the specimen volume, bulk tensile properties and Charpy impact energies remained effectively unchanged across irradiation doses. However, fracture strain exhibited a modest downward trend, declining by ~4.8% in Class 1 and ~2.4% in Class 2, consistent with incipient ductility loss from irradiation-induced defect formation. Nanoindentation revealed clear hardening in both steel grades: Class 2 maintained higher absolute hardness than Class 1 in all conditions, but Class 1 experienced a larger relative hardness increase (20.4% vs. 15.4%). Reduced modulus measurements fluctuated non-monotonically, indicating that elastic stiffness is less directly governed by defect accumulation than plastic resistance. STEM–BF imaging and SAED analysis corroborated these mechanical findings, showing higher defect densities and greater lattice distortion in irradiated Class 1 specimens compared to Class 2. To comprehensively capture irradiation effects across multiple length scales, future work should integrate nanoindentation with additional micro-mechanical tests—such as micro-pillar compression, micro-cantilever bending, and micro-tensile testing—to quantify local yield strength, fracture toughness, and strain localization within the ion-damaged zone. This multi-scale approach will deepen understanding of irradiation hardening and embrittlement mechanisms in reactor pressure vessel steels. To comprehensively capture irradiation effects across multiple length scales, future work will integrate nanoindentation with FIB-fabricated micro-mechanical assays—including micro-pillar compression, micro-cantilever bending, and micro-tensile testing—performed directly within the ion-damaged region. These localized tests will enable precise quantification of yield strength, fracture toughness, and strain localization in the shallow (~2.2 µm) damage layer, thereby deepening understanding of irradiation hardening and embrittlement mechanisms in reactor pressure vessel steels. Additionally, subsequent studies will incorporate quantitative microstructural analyses such as EBSD-derived grain size distributions, dislocation density measurements, and phase fraction determinations to directly correlate microstructural features with mechanical performance.

## Data Availability

The original contributions presented in this study are included in the article. Further inquiries can be directed to the corresponding author.
